# Breastfeeding – a survey of fathers’ support needs and preferred sources of information

**DOI:** 10.1186/s13006-024-00654-9

**Published:** 2024-07-17

**Authors:** Kidane Tadesse Gebremariam, Karen Wynter, Miaobing Zheng, Jonathan Charles Rawstorn, Elizabeth Denney-Wilson, Rachel Laws

**Affiliations:** 1 Institute for Physical Activity and Nutrition, School of Exercise and Nutrition Sciences, Deakin University, Melbourne, Australia; 2 School of Public Health, Mekelle University, Mekelle, Ethiopia; 3 School of Health and Social Development, Faculty of Health, Deakin University, Melbourne, Australia; 4 Department of Psychiatry, School of Clinical Sciences, Monash University, Melbourne, Australia; 5 School of Nursing and Midwifery, Deakin University, Melbourne, Australia; 6 NHMRC Centre of Research Excellence in Digital Technology to Transform Chronic Disease Outcomes, Melbourne, Australia; 7 Susan Wakil School of Nursing and Midwifery, University of Sydney, Sydney, Australia

**Keywords:** Fathers, Breastfeeding, Partner, Fatherhood

## Abstract

**Background:**

Fathers can be a critical source of breastfeeding support for their partner, but little is known about what fathers would like to learn about breastfeeding. Partner’s support and encouragement enhances mother’s breastfeeding confidence and boost the capacity to address breastfeeding difficulties effectively. The aims of this study were to explore what fathers regard as important to learn around breastfeeding, and their current and preferred sources of information.

**Methods:**

A structured online survey was conducted, between September 2022 and November 2022, with fathers containing three sections: (1) sociodemographic variables; (2) perceived importance of 26 breastfeeding topics; and (3) sources of breastfeeding information. A convenience sample of expectant and current fathers aged 18 years or older, who were expecting a baby or had a child aged one year or younger, living in Australia, and able to complete survey in English was recruited. Participants were recruited on Facebook advertisement.

**Results:**

A total of 174 fathers participated in the study, majority (75%) were aged 30–39 years, current dads (74%), and university educated (69%). The breastfeeding topics that fathers perceived as the most important/ important to learn about were how to work with their partner to overcome breastfeeding challenges, how fathers can be involved with their breastfed baby, the types of support fathers can provide to breastfeeding mothers, what to expect in the first week and the benefits of breastfeeding. The most preferred health professional sources of breastfeeding information were midwives, child and family nurses and doctors. Among non-health professional sources of support, mobile app, friends and family were most popular.

**Conclusion:**

Breastfeeding information to enhance fathers’ knowledge and awareness of common breastfeeding challenges, and fathers’ role in supporting their breastfeeding partner, appear to be (most) important for fathers. Mobile app appears to be among the most preferred non-health professional ways to provide breastfeeding information to fathers.

## Background

Adequate support for lactating mothers is crucial to improve exclusive breastfeeding [1] as unsupported mothers are less likely to initiate and continue breastfeeding [2]. Fathers can be an important source of support as their beliefs on whether their partners should breastfeed can predict maternal intention to breastfeed [3]. According to the Australian Infant Feeding Survey, 22% of mothers indicated their partners’ encouragement as one of the reasons for breastfeeding [4]. Among breastfeeding mothers, higher levels of partner support and encouragement have been shown to increase breastfeeding confidence [5] and improve ability to solve breastfeeding-related challenges [6].

Health education programs designed for fathers during the prenatal or postnatal period have the potential to improve fathers’ involvement in breastfeeding, provide support to their breastfeeding partners and positively influence breastfeeding outcomes [7–9]. Qualitative studies have found that available breastfeeding information does not adequately address fathers’ needs on how to provide breastfeeding support and overcome breastfeeding-related problems [10].

Fathers receive breastfeeding information from diverse sources including health care professionals, printed materials/resources, antenatal support groups, the internet, and other technologies [11]. Regarding support received from health care professionals, fathers indicate that they are overwhelmed with the amount of information they receive [12]. In addition, fathers are disappointed with the lack of support specifically targeting fathers [10, 13] and fathers can feel left out [14]. Although fathers want to learn how to support their breastfeeding partner [15, 16], they have indicated that they have limited access to support services due to their working hours [17, 18]. In line with this, research has shown that it is difficult for face-to-face support services to reach fathers during the perinatal period; thus, digital sources of breastfeeding support could improve engagement [19]. Fathers also report that the internet is an important source of breastfeeding support [20, 21]. Peer support from experienced fathers appears to be one of the sources of breastfeeding social support for fathers and can help them improve their confidence [12, 15].

Despite the importance of support from women’s partners in improving breastfeeding outcomes, few interventions have been directed at fathers [22, 23]. Existing interventions have showed significant improvement in fathers’ knowledge, attitude and awareness of breastfeeding. For instance, an intervention which involved providing information face to face to 72 couples in groups in combination with written materials. The results showed an increase in rates of exclusive breastfeeding at 24 weeks, duration of breastfeeding when infants were 4 and 6 months, and fathers’ knowledge and attitude towards breastfeeding compared to the control group [24]. Similarly, a face to face and written material intervention including 50 fathers found improved breastfeeding awareness in the intervention compared to the control group [25]. An intervention involving face to face, written materials, mass media and public events in their intervention to provide breastfeeding information to fathers, and demonstrated improved rates of exclusive breastfeeding at 24 weeks [26]. Other interventions delivering breastfeeding intervention to couples using face to face, written material, telephone, email and websites [9] improved duration of breastfeeding at 12 months, breastfeeding self-efficacy and partner breastfeeding support.

Mobile health (mHealth) interventions providing postnatal parenting support, including breastfeeding support for fathers, have shown good uptake and acceptance [19, 27, 28]. In the Milk Man Mobile App intervention, fathers indicated that the Milk Man app was an acceptable source of breastfeeding information [29]. A co-parenting mHealth pre-post study improved fathers’ breastfeeding knowledge, attitude and self-efficacy [30]. Moreover, SMS-based breastfeeding intervention involving fathers in a low-income country improved exclusive breastfeeding in the first three months of children’s life compared to the control group [31].

Most of the face-to-face or mHealth interventions were designed by researchers and provided to fathers as resources; however, it is important to involve fathers throughout the design process [32] and create an active collaboration to help address their needs and identify suitable strategies [33]. Thus, the purpose of this study was to explore expectant and current fathers’ breastfeeding information and support needs as part of the design of a breastfeeding mobile app for fathers.

## Methods

### Design

This study was a cross sectional online survey conducted between September 2022 and November 2022.

### Participants and recruitment

Eligible participants were fathers aged 18 years or older, who were expecting a baby or had a child aged one year or younger, living in Australia and with sufficient English language ability to complete the survey. A convenience sample was recruited via Facebook advertisements that targeted fathers using **Interests**: fatherhood, parenting or family; and **Parents**: parents (up to 12 months). Facebook send the advertisement to potential participants, the advertise appear in their Facebook page when accessing Facebook. The Facebook advertisement reached 145,569 potential participants across Australia with 671 clicking the advertisement link $0.75 USD per click ($500 AUD). Eligible participants were provided with a plain language statement describing details of the study and asked to provide informed consent online.

### Data collection

After providing consent, fathers were directed to an online survey, hosted on Qualtrics. Fathers were asked to rate the importance of 26 breastfeeding topics categorised under five domains: (1) deciding how to feed your baby; (2) practicalities of breastfeeding; (3) breastfeeding during the early days; (4) breastfeeding challenges/concerns; and (5) breastfeeding partner support; rated on a five-point Likert scale ranging from 1 (Not important at all) to 5 (Most important). Questions were adapted from a previous survey which contained 61 topics [30] and the research team modified the topics based on their research experience and expertise. The second part was about fathers’ current, and preferred sources of breastfeeding support. Lastly, Fathers were asked to provide their sociodemographic characteristics.

### Data analysis

Data were exported from Qualtrics and analysed using IBM SPSS Statistics 29.0. After data cleaning and coding, descriptive statistics were conducted. Sociodemographic characteristics, topic importance, and current and preferred information sources are presented using frequencies and percentages.

## Results

Out of 289 eligible participants who consented to participate and started the survey, 174 (60%) completed the survey. The majority were born in Australia (79%), English speaking (93%), working full-time (84%), and university educated (70%). The majority were current fathers (83%); very few (17%) were expecting their first baby (Table 1). The age of children rage from 2-days to 1-year, with mean age of 6 months with a standard deviation of *±* 4.6 months.

**Table 1:** Participant characteristics (*n* = 174)

Fathers’ characteristics	*n* (%)
**Age**	
25–29	18 (10.3)
30–34	73 (42)
35–39	59 (33.9)
40+	23 (13.2)
Prefer not to answer	1 (0.6)
Country of Birth	
Australia	138 (79.3)
Other	36 (20.7)
Main language spoken at home	
English	163 (93.7)
Other	11 (6.3)
Status	
Current dad	129 (74.2)
Current dad and expecting baby	15 (8.6)
Expectant -first baby	30 (17.2)
First baby	
Yes	116 (66.7)
No	58 (33.3)
Highest education level	
High school education or less	14 (8)
Trade certificate or diploma	37 (21.3)
University degree or higher	121 (69.5)
Preferred not to answer	1 (0.6)
Working status	
Working full-time	146 (83.9)
Working part-time/casual	18 (10.3)
Unemployed	2 (1.1)
Keeping house /raising children full-time	3 (1.7)
Studying full or part-time	3 (1.7)

### Breastfeeding topics

Based on the score, the top ten breastfeeding topics perceived to be most important were: Breastfeeding partner support: how to work with your partner to overcome breastfeeding challenges (98.3%), how fathers/partners can be involved with their breastfed baby (98.3%) and type of support fathers/partners can provide to breastfeeding mothers (98.3%); Deciding how to feed your baby: benefits of breastfeeding (97.7%); Breastfeeding during early days: what to expect in the first week (97.7) and what to expect in the first 6 weeks (97.1); Breastfeeding challenges/concerns: breastfeeding if mum is sick or taking medication (96%), what to do if baby refuses breast (95.4) and understanding if baby is growing well (94.8%), and Practicalities of breastfeeding: knowing when baby is hungry or full (95.4%) (Table 2).

**Table 2:** Breastfeeding topics (*N* = 174)

	Most important/ important	Neutral	Unimportant/not important at all
**Deciding how to feed your baby**			
Benefits of breastfeeding	170 (97.7)	2 (1.1)	2 (1.2)
Difference b/n BF and formula	161 (92.5)	11 (6.3)	2 (1.2)
How long to breastfeed	146 (83.9)	25 (14.4)	3 (1.7)
**Practicalities of breastfeeding**			
How often to breastfeed	161 (92.5)	12 (6.9)	1 (0.6)
Knowing when baby is hungry or full	166 (95.4)	7 (4)	1 (0.6)
Knowing if baby is getting enough mink	164 (94.3)	9 (5.2)	1 (0.6)
Position of breastfeeding	133 (76.4)	28 (16.1)	12 (6.9)
Pumping and storing expressed milk	154 (88.5)	18(10.3)	1 (0.6)
**Breastfeeding during early days**			
What to expect in the first week	170 (97.7)	3 (1.7)	1 (0.6)
What to expect in the first 6 weeks	169 (97.1)	15 (8.6)	1 (0.6)
Skin to skin contact	157 (90.2)	15 (8.6)	2 (1.1)
**Breastfeeding challenges/concerns**			
What to do if baby refuses breast	166 (95.4)	7 (4)	1 (0.6)
Help your partner manage sore nipples	150 (86.2)	20 (11.5)	4 (2.3)
How to increase breastmilk supply	144 (82.8)	26 (14.9)	4 (2.3)
Breastfeeding a sleepy baby	123 (70.7)	44 (25.3)	7 (4)
Managing fast flow of breastmilk	109 (62.6)	54 (31)	11 (6.3)
Breastfeeding twins/multiples	69 (39.7)	76 (43.7)	29 (16.7)
Understanding if baby is growing well	165 (94.8)	9 (5.2)	
Breastfeeding if mum is sick or taking medication	167 (96)	7 (4)	
Managing mixed feeding (feeding both breastmilk and formula)	148 (85.1)	22 (12.6)	4 (2.2)
Substance use while breastfeeding – alcohol, recreational drugs or cigarettes	146 (83.9)	23 (13.2)	5 (2.8)
**Breastfeeding partner support**			
How to work with your partner to overcome breastfeeding challenges	171 (98.3)	3 (1.7)	
How fathers/partners can be involved with their breastfed baby	171 (98.3)	2 (1.1)	1 (0.6)
Type of support fathers/partners can provide to breastfeeding mothers	171 (98.3)	3 (1.7)	
Where to getting help with breastfeeding	159 (91.4)	12 (6.9)	3 (1.7)
How to set breastfeeding goals with your partner	119 (68.4)	45 (25.9)	10 (5.8)

Table 3 presents fathers’ sources of breastfeeding information and perceived helpfulness. Most fathers reported they received breastfeeding information from midwives (82%). More than half of the fathers accessed information from child and family nurses (58%) or doctors (55%). Fathers also received breastfeeding support from a range of non-health professional sources; most commonly Google (47.7%), friends (43.7) and family (43.7). Most fathers rated the perceived helpfulness of the support from health professionals as very beneficial/beneficial, but the proportion varied between specific sources (e.g., Australia Breastfeeding association = 90%; doctors = 65%). Non-health professional sources of support were less frequently rated as very beneficial/beneficial (friends = 58%; mobile app = 57%). On the other hand, social media, online parenting forums, and Google were the most likely to be rated as only moderately/somewhat helpful (Table 3).

**Table 3:** Current sources of breastfeeding information and perceived helpfulness (*N* = 174)

	Received advice		Very beneficial/ beneficial	Moderately beneficial/ somewhat beneficial	Not beneficial at all
**Health professional**					
Midwife	144 (82.7)		118 (81.9)	24 (16.7)	2 (1.4)
Child and family nurse	102 (58.6)		87 (85.3)	13 (12.7)	2 (2)
Doctor	96 (55.2)		63 (65.6)	29 (30.2)	4 (4.2)
Antenatal classes	72 (41.4)		48 (66.6)	23 (32)	1 (1.4)
Australia Breastfeeding Association (ABA)	62 (35.6)		56 (90.3)	6 (9.7)	
Practicing nurse	40 (23)		30 (75)	8 (20)	2 (5)
**Non health professional**					
Google	83 (47.7)		30 (36.1)	53 (63.9)	
Friends	76 (43.7)		44 (57.9)	31 (40.8)	1 (1.3)
Family	76 (43.7)		39 (51.3)	36 (47.4)	1 (1.3)
Social media	35 (20.1)		9 (25.7)	24 (68.6)	2 (5.7)
Mobile app	28 (16.1)		16 (57.1)	11 (39.3)	1 (3.6)
Online Parents’ Forum	27 (15.5)		7 (25.9)	20 (74.1)	

Fathers indicated that the top three preferred sources of breastfeeding information from health professionals are midwife, child and family nurse, and doctor. For the non-health professional sources of breastfeeding information, fathers rated mobile app, friends and families as their top three sources. Regarding preferred sources of breastfeeding information, only 3.4% of participants indicated they didn’t need any information (Table 4).

**Table 4:** Preferred sources of breastfeeding information (*N* = 174)

Preferred source of BF support	*N* (%)
Health professional	
Midwife	118 (67.8)
Child and Family Nurse	95 (54.6)
Doctor	70 (40.2)
Australia Breastfeeding Association (ABA)	64 (36.8)
Antenatal Classes	56 (32.2)
Practice Nurse	40 (23)
Non health professional	
Mobile App	57 (32.8)
Friends	29 (16.6)
Family	23 (13.2)
Google	21 (12.1)
Online Parents Forum	16 (9.2)
Social Media	6 (3.4)
I don’t need any information	6 (3.4)
Other	7 (4)

The majority, 140 (80%) of fathers thought that a mHealth app could be useful to provide breastfeeding information, with preferred information delivery methods for an mHealth app being videos (58%), written articles (55%), links to other sources of information (55%), forum with dads (40%), push notifications (35%), quizzes (23.6%) and SMS (14.3%).

## Discussion

This paper explored the areas where fathers needed help to provide breastfeeding support to their partner, and current and preferred information sources of breastfeeding information. Fathers indicated that they need breastfeeding support across all domains examined in this survey: deciding how to feed a baby, practicalities of breastfeeding; breastfeeding during the early days; ways to overcome breastfeeding challenges; and how to provide practical breastfeeding support to their partner. Fathers indicated that they preferred health professional sources of breastfeeding support from midwifes child and family nurses and doctors; and preferred non-health professionals’ sources include mobile app, friends and family.

In line with previous studies, the current study showed that fathers were keen to receive information to improve their understanding of the benefits of breastfeeding. Previous studies reported that fathers were unable to clearly explain the benefits of breastfeeding and had low breastfeeding knowledge [17, 18, 34]. In line with this, fathers in the current study indicated that the benefit of breastfeeding is an important topic to learn about. Fathers who received health education on the benefits of breastfeeding and providing practical support to their breastfeeding partners were more involved in supporting their breastfeeding partners, which improved rates of exclusive breastfeeding at 4 months [35]. Previous studies showed fathers had a lack of understanding on the practicalities of breastfeeding, for example they had difficulty determining if the baby was hungry or full [18, 34]. Similarly, fathers in the current study reported that information regarding knowing when baby is hungry or full is crucial for them to provide breastfeeding support to their partner.

Previous studies indicated that provision of breastfeeding information can help fathers to overcome breastfeeding challenges and provide better support to their partner [8, 36]. In line with a previous study [30], fathers in this study indicated the importance of information related to breastfeeding in the first week, and breastfeeding in the first six weeks. In addition, fathers in the current study showed that they were concerned about infant growth in the early days. Similarly, a previous study showed that following birth, fathers were concerned about baby’s weight which was a major reason for moving from breastfeeding to formula [17]. Moreover, breast refusal is a challenge for mothers which is caused either by infant-related factors such as infection and distraction during breastfeeding, or by mother-related factors, which can lead to changes in milk taste and supply, including a mother changing perfume, mastitis, mothers being unhealthy, depressed or taking medications [37]. Consistent with this finding, fathers in the current study also identified baby breast refusal as a challenge to continued breastfeeding.

The support for men during the transition to fatherhood is important in creating good relationships with their partner and the new child [28]. Fathers in the current study showed knowing how to be involved with their breastfed baby is among the breastfeeding topics they are interested to learn about. Similarly, fathers were happy with receiving information related to providing breastfeeding support to their partner, and how to get involved with the baby [38]. Fathers can provide support to their breastfeeding partner by learning and sharing knowledge about the benefits and expressing appreciation for their partner’s willingness to breastfeed the baby, sharing housework and childcare [18, 39]. Fathers in the current study indicated that it is important to know how fathers could work with their breastfeeding partner. Previous research supports this and suggests that infant feeding support should consider the relationship between parents, including their ability to work as a team [40, 41].

In the present study fathers indicated that they had received breastfeeding support from both health professionals and non-health professional sources; however, perceived benefits differed. While professional sources were frequently rated as very beneficial, particularly specialist sources like midwife, child and family nurses and Australian Breastfeeding Association, non-professional sources were more likely to be rated as only moderately beneficial. A previous study showed that general health professionals such as GPs lack knowledge and conviction to provide breastfeeding support, thus, they need better training [42]. Breastfeeding education for GPs has been found to improve knowledge and provision of proactive breastfeeding support [37, 43]. Fathers receive breastfeeding support from different sources [11] including face-to-face from health professionals [12] and peer support from experienced fathers [12, 15]. The challenges accessing face-to-face services during working hours can reduce fathers’ ability to accompany their partners during postnatal visits [17, 18] and often the services are directed at mothers [44].

While the internet has been reported to be among the most important sources of breastfeeding information for fathers [20, 21] and Google was the most frequently used non-professional source of support in the current study, it was substantially more likely to be perceived as only moderately or somewhat beneficial. When fathers were asked about non-professional source of breastfeeding support, mobile apps were perceived to be most helpful alongside information and support from family and friends. Moreover, 80% of the participants indicated their interest to receive breastfeeding information via mobile app. mHealth apps developed by health professionals are considered as credible source of information with better trustworthiness than commercially developed apps [45, 46], and commercially developed pregnancy-related apps incorporating breastfeeding have been found to be poor quality in terms of appropriateness of the breastfeeding content and guidelines [47]. Although few in number, mHealth breastfeeding interventions targeting fathers in Australia [29], Canada [30], and Ethiopia [48] have showed high levels of acceptability and improvement in breastfeeding knowledge, attitude and self-efficacy amongst fathers.

Breastfeeding interventions in the future should include the participation of fathers, whether provided by healthcare professionals or non-healthcare professionals. It is important to involve parents in the design and development process of these interventions, allowing them to contribute their opinions on the intervention’s content. Furthermore, future research should consider the perspective of mothers regarding the involvement of fathers in breastfeeding. Additionally, interventions aimed at promoting breastfeeding should prioritize the concept of coparenting, fostering teamwork between parents to help them achieve their breastfeeding goals.

The current study has strengths and limitations that should be considered when interpretating the findings. This study is one of the first studies exploring the breastfeeding information needs specific for fathers. However, there are also limitations to consider. The convenience nature of study participant recruitment and small sample size contributed to a homogenous sample of highly educated and predominantly Australian-born fathers, which may influence the generalisability of the findings. In addition, as majority of the participants were current fathers, the perspectives of first-time expectant fathers may not be fully represented in the study. It is also worth noting that the study did not include individuals of the same sex, so caution must be exercised when applying the findings to this population.

## Conclusion

Fathers indicated the breastfeeding topics they preferred to learn were mostly on deciding how to feed a baby, practicality of breastfeeding, breastfeeding during early days, breastfeeding challenges, how they can best support their breastfeeding partners. Fathers showed interest in the future to access both professional and non-professional sources of breastfeeding support. Mobile apps appear to be the preferred non health professional source of information for fathers on breastfeeding while preferred health professional sources include midwives and child and family nurses. The findings of this study will contribute to the content development of breastfeeding mobile app for fathers.

## Data Availability

The datasets used and/or analysed during the current study are available from the corresponding author on reasonable request.

## References

[1] WHO (2001). Global strategy for infant and young child feeding. The optimal duration of exclusive breastfeeding.

[2] Dennis CL (2002). Breastfeeding initiation and duration: a 1990–2000 literature review. J Obstet Gynecol Neonatal Nurs.

[3] Scott JA, Landers MC, Hughes RM, Binns CW (2001). Factors associated with breast feeding at discharge and duration of breast feeding. J Pediatr Child Health.

[4] Australian Institute of Health and Welfare. 2010 Australian national infant feeding survey: indicator results. In. Canberra: AIHW; 2011.

[5] Hauck Y, Hall WA, Jones C (2007). Prevalence, self-efficacy and perceptions of conflicting advice and self-management: effects of a breastfeeding journal. J Adv Nurs.

[6] Mannion CA, Hobbs AJ, McDonald SW, Tough SC (2013). Maternal perceptions of partner support during breastfeeding. Int Breastfeed J.

[7] Maycock B, Binns CW, Dhaliwal S, Tohotoa J, Hauck Y, Burns S, Howat P (2013). Education and support for fathers improves breastfeeding rates: a randomized controlled trial. J Hum Lactation.

[8] Pisacane A, Continisio GI, Aldinucci M, D’Amora S, Continisio P (2005). A controlled trial of the father’s role in breastfeeding promotion. Pediatrics.

[9] Abbass-Dick J, Stern SB, Nelson LE, Watson W, Dennis CL (2015). Coparenting breastfeeding support and exclusive breastfeeding: a randomized controlled trial. Pediatrics.

[10] Tohotoa J, Maycock B, Hauck YL, Howat P, Burns S, Binns CW (2009). Dads make a difference: an exploratory study of paternal support for breastfeeding in Perth, Western Australia. Int Breastfeed J.

[11] Sihota H, Oliffe J, Kelly MT, McCuaig F (2019). Fathers’ experiences and perspectives of breastfeeding: a scoping review. Am J Men’s Health.

[12] Ayton J, Hansen E (2016). Complex young lives: a collective qualitative case study analysis of young fatherhood and breastfeeding. Int Breastfeed J.

[13] Banks E, Killpack S, Furman L (2013). Low-income inner-city fathers and breastfeeding–where’s the program for us?. Breastfeed Med.

[14] Sherriff N, Hall V, Panton C (2014). Engaging and supporting fathers to promote breast feeding: a concept analysis. Midwifery.

[15] Brown A, Davies R (2014). Fathers’ experiences of supporting breastfeeding: challenges for breastfeeding promotion and education. Maternal Child Nutr.

[16] Rempel LA, Rempel JK, Moore KCJ (2017). Relationships between types of father breastfeeding support and breastfeeding outcomes. Maternal Child Nutr.

[17] Sherriff N, Hall V (2011). Engaging and supporting fathers to promote breastfeeding: a new role for Health visitors?. Scand J Caring Sci.

[18] Sherriff N, Hall V, Pickin M (2009). Fathers’ perspectives on breastfeeding: ideas for intervention. Br J Midwifery.

[19] White BK, Giglia RC, Scott JA, Burns SK (2018). How new and expecting fathers engage with an app-based online forum: qualitative analysis. JMIR Mhealth Uhealth.

[20] Mitchell-Box K, Braun KL (2012). Fathers’ thoughts on breastfeeding and implications for a theory-based intervention. J Obstet Gynecol Neonatal Nurs.

[21] Bennett AE, McCartney D, Kearney JM (2016). Views of fathers in Ireland on the experience and challenges of having a breast-feeding partner. Midwifery.

[22] Abbass-Dick J, Brown HK, Jackson KT, Rempel L, Dennis C-L (2019). Perinatal breastfeeding interventions including fathers/partners: a systematic review of the literature. Midwifery.

[23] Tadesse K, Zelenko O, Mulugeta A, Gallegos D (2018). Effectiveness of breastfeeding interventions delivered to fathers in low- and middle-income countries: a systematic review. Maternal Child Nutr.

[24] Su M, Ouyang YQ (2016). Father’s role in Breastfeeding Promotion: lessons from a quasi-experimental trial in China. Breastfeed Med.

[25] Raeisi K, Shariat M, Nayeri F, Raji F, Dalili H (2014). A single center study of the effects of trained fathers’ participation in constant breastfeeding. Acta Medica Iranica.

[26] Bich TH, Hoa DT, Målqvist M (2014). Fathers as supporters for improved exclusive breastfeeding in Viet Nam. Matern Child Health J.

[27] Fletcher R, May C, Wroe J, Hall P, Cooke D, Rawlinson C, Redfern J, Kelly B (2016). Development of a set of mobile phone text messages designed for new fathers. J Reproductive Infant Psychol.

[28] Fletcher R, Kay-Lambkin F, May C, Oldmeadow C, Attia J, Leigh L (2017). Supporting men through their transition to fatherhood with messages delivered to their smartphones: a feasibility study of SMS4dads. BMC Public Health.

[29] White B, Giglia RC, White JA, Dhaliwal S, Burns SK, Scott JA (2019). Gamifying breastfeeding for fathers: process evaluation of the milk man mobile app. JMIR Pediatr Parent.

[30] Abbass-Dick J, Xie F, Koroluk J, Alcock Brillinger S, Huizinga J, Newport A, Goodman WM, Dennis CL (2017). The development and piloting of an eHealth breastfeeding resource targeting fathers and partners as co-parents. Midwifery.

[31] Gebremariam KT, Mulugeta A, Gallegos D (2023). Theory-based mHealth targeting fathers and mothers to improve exclusive breastfeeding: a quasi-experimental study. Int Breastfeed J.

[32] Sanders EBN, Stappers PJ (2008). Co-creation and the new landscapes of design. CoDesign.

[33] Visser FS, Stappers PJ, van der Lugt R, Sanders EBN (2005). Contextmapping: experiences from practice. CoDesign.

[34] Henderson L, McMillan B, Green JM, Renfrew MJ (2011). Men and infant feeding: perceptions of embarrassment, sexuality, and social conduct in white low-income British men. Birth.

[35] Panahi F, Rashidi Fakari F, Nazarpour S, Lotfi R, Rahimizadeh M, Nasiri M, Simbar M (2022). Educating fathers to improve exclusive breastfeeding practices: a randomized controlled trial. BMC Health Service Res.

[36] Swanson V, Power KG (2005). Initiation and continuation of breastfeeding: theory of planned behaviour. J Adv Nurs.

[37] Burt S, Whitmore M, Vearncombe D, Dykes F (2006). The development and delivery of a practice-based breastfeeding education package for general practitioners in the UK. Maternal Child Nutr.

[38] Abbass-Dick J, Dennis CL (2018). Maternal and paternal experiences and satisfaction with a co-parenting breastfeeding support intervention in Canada. Midwifery.

[39] Rempel LA, Rempel JK (2011). The breastfeeding team: the role of involved fathers in the breastfeeding family. J Hum Lactation.

[40] Sahip Y, Turan JM (2007). Education for expectant fathers in workplaces in Turkey. J Biosoc Sci.

[41] Susin LR, Giugliani ER (2008). Inclusion of fathers in aniIntervention to promote breastfeeding: impact on breastfeeding rates. J Hum Lactation.

[42] Holtzman O, Usherwood T (2018). Australian general practitioners’ knowledge, attitudes and practices towards breastfeeding. PLoS ONE.

[43] Holmes AV, McLeod AY, Thesing C, Kramer S, Howard CR (2012). Physician breastfeeding education leads to practice changes and improved clinical outcomes. Breastfeed Med.

[44] Mithani Y, Premani ZS, Kurji Z, Rashid S (2015). Exploring fathers’ role in breastfeeding practices in the urban and semiurban settings of Karachi, Pakistan. J Perinat Educ.

[45] Gebremariam KT, Zelenko O, Hadush Z, Mulugeta A, Gallegos D (2020). Could mobile phone text messages be used for infant feeding education in Ethiopia? A formative qualitative study. Health Inf J.

[46] Peng W, Kanthawala S, Yuan S, Hussain SA (2016). A qualitative study of user perceptions of mobile health apps. BMC Public Health.

[47] Musgrave LM, Kizirian NV, Homer CSE, Gordon A (2020). Mobile phone apps in Australia for improving pregnancy outcomes: systematic search on App stores. JMIR Mhealth Uhealth.

[48] Gebremariam KT, Mulugeta A, Gallegos D (2023). Co-design and implementation of a mHealth intervention targeting fathers and mothers to improve breastfeeding. BMC Med Inf Decis Mak.

